# Virulomic Analysis of Multidrug-Resistant *Klebsiella pneumoniae* Isolates and Experimental Virulence Model Using *Danio rerio* (Zebrafish)

**DOI:** 10.3390/antibiotics11111567

**Published:** 2022-11-07

**Authors:** Edson Luiz Tarsia Duarte, Camila Fonseca Rizek, Evelyn Sanchez Espinoza, Ana Paula Marchi, Saidy Vasconez Noguera, Marina Farrel Côrtes, Bianca H. Ventura Fernandes, Thais Guimarães, Claudia M. D. de Maio Carrilho, Lauro V. Perdigão Neto, Priscila A. Trindade, Silvia Figueiredo Costa

**Affiliations:** 1 Medical Investigation Laboratory (LIM49), Tropical Medicine Institute of University of São Paulo, Av. Dr. Eneas Carvalho de Aguiar, 470, São Paulo 05403-000, Brazil; 2 Technical Division of Teaching and Research Support—Zebrafish Unit, Faculty of Medicine of the University of São Paulo Biotherism Center, Av. Dr. Arnaldo, 455, São Paulo 01246-903, Brazil; 3 Hospital Infection Control Commission, Hospital das Clinicas of Faculty of Medicine, University of São Paulo (HC-FMUSP), Dr. Eneas Carvalho de Aguiar 255, São Paulo 05403-000, Brazil; 4 Hospital Infection Control Commission of Londrina’s University Hospital (HU-UEL), Av. Robert Koch, 60, Londrina 86038-350, Brazil; 5 Health Sciences Center, Clinical and Toxicological Analysis Department, Federal University of Santa Maria (UFSM), Av. Roraima, 1000, Prédio 26, Camobi, Santa Maria 97105-900, Brazil

**Keywords:** WGS, zebrafish, *K. pneumoniae*, virulence factor, MDR, MLST, sequence type, ST16

## Abstract

This study evaluates a possible correlation between multidrug-resistant *Klebsiella pneumoniae* strains and virulence markers in a *Danio rerio* (zebrafish) model. Whole-genome sequencing (WGS) was performed on 46 strains from three Brazilian hospitals. All of the isolates were colistin-resistant and harbored bla_KPC-2_. Ten different sequence types (STs) were found; 63% belonged to CC258, 22% to ST340, and 11% to ST16. The virulence factors most frequently found were type 3 fimbriae, siderophores, capsule regulators, and RND efflux-pumps. Six strains were selected for a time-kill experiment in zebrafish embryos: infection by ST16 was associated with a significantly higher mortality rate when compared to non-ST16 strains (52% vs. 29%, *p* = 0.002). Among the STs, the distribution of virulence factors did not differ significantly except for ST23, which harbored a greater variety of factors than other STs but was not related to a higher mortality rate in zebrafish. Although several virulence factors are described in *K. pneumoniae*, our study found ST16 to be the only significant predictor of a virulent phenotype in an animal model. Further research is needed to fully understand the correlation between virulence and sequence types.

## 1. Introduction

*Klebsiella pneumoniae* is one of the bacterial pathogens declared as an “urgent threat” by healthcare organizations over the world and can be related to community-acquired and nosocomial infections [1]. *K. pneumoniae*’s great capacity for acquiring genetic material has led to increasingly higher rates of antibiotic resistance, with several reports of drug-resistant and multidrug-resistant (resistant to three or more antimicrobial classes) isolates across the world. This scenario is aggravated when virulence is considered [2,3,4].

*Klebsiella pneumoniae* virulence can be related to a wide range of factors, from lipopolysaccharides and O antigen to siderophores and fimbriae. Searching for specific virulence factors or serotypes can be misleading for understanding the microorganism’s true virulence capacity since serotypes related to hypervirulence were also observed to be related to classic *K. pneumoniae* strains (cKp) [3,5]. Although the evolution of molecular techniques has allowed virulence to be more widely studied on a genetic level for the past two decades, a clear correlation between gene, gene expression, and in vivo significance is yet to be stablished.

Few authors have used the *Danio rerio* (zebrafish) model for assessing the immune response of infection caused by *Enterobacteriaceae*, such as *Salmonella*, *Shigella*, and *Klebsiella* [6,7,8]. Regarding specific virulence evaluation, the literature is very modest—few studies used the zebrafish model to compare *K. pneumoniae* strains virulence [3], and most of them analyzed specific virulence factors [9]. Moreover, methods were not standardized among the few publications that employed the zebrafish model on *K. pneumoniae* studies, especially regarding inoculum properties, and different studies used different routes of inoculation and different bacterial inoculum, which ranged from 10^2^ to 10^8^ CFU/mL [3,8,9,10].

Here, we intended to analyze the genotypic and in vivo virulence of clinical multidrug-resistant *Klebsiella pneumoniae* and the possible correlation between virulence markers and mortality in a zebrafish model.

## 2. Results

### 2.1. WGS, Resistance and Virulence Genes

All 46 strains harbored *bla*_KPC-2_. Colistin resistance was primarily due to chromosomal mutations as *Ipx*M, *yci*M, *pmr*B, *ept*A (*pmr*C), *crr*B, and *mgr*B were the most common mutated genes. The *mcr*-1 gene was present in five strains (11%); all of these isolates were from the bone marrow transplant unit of Hospital das Clínicas da Faculdade de Medicina de São Paulo (HC-FMUSP) and belonged to different sequence types (STs) (one ST11, one ST16, two ST101, one ST437). All strains harbored at least one beta-lactamase other than KPC-2, most commonly SHV-11 (74%), TEM-1B (67%), and CTX-M-15 (33%). Fosfomycin resistance related to gene *fos*A was present in all isolates. In total, ten STs were identified, and four of them (ST11, ST25, ST258, and ST437) can be grouped in clonal complex 258 (CC258), which was the most frequent among isolates (63%).

The most common genes related to virulence found across all strains are presented in Table 1. Six strains were selected for the model, hereby identified by the letters A (strain 4223), B (strain 4290), C (strain 5921), D (strain 4449), E (strain 4553), and F (strain 5919).

**Figure 1:**
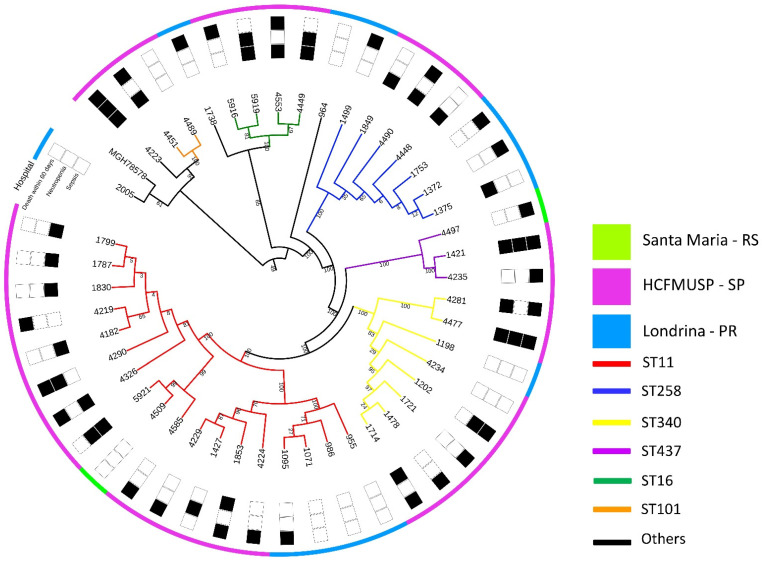
Phylogenetic tree of all 46 *K. pneumoniae* isolates in this study, based on single-nucleotide polymorphisms (SNPs) of core genome. Colors on the outermost circle represent place of origin, while branch colors represent sequence types. Each number represents one strain, with each isolated from a different patient.

An SNP tree depicts the phylogenetic relation between all isolates included in the study in Figure 1, and characteristics of the six strains selected for the zebrafish model can be seen in Table 1.

### 2.2. Zebrafish Virulence Model Preliminary Phase: Determination of Inoculum

Mortality was 100% at 24 h for the group that received a 10^9^ CFU/mL inoculum and at 48 h for the group that received a 10^8^ CFU/mL inoculum. Mortality in the groups that received concentrations of 10^2^, 10^4^, and 10^6^ CFU/mL was 26%, 29%, and 63%, respectively. Cardiotoxicity, defined as the development of cardiac edema, was observed in 13% of embryos injected with an inoculum concentration of 10^6^ CFU/mL but not in the groups that received concentrations of 10^4^ CFU/mL and lower. No concentrations under 10^6^ CFU/mL produced teratogenic effects; from 10^8^ CFU/mL and higher, it was not possible to evaluate malformations due to 100% lethality observed in the first 48 h.

Hence, we established the optimal concentration for the experiment to be 10^6^ CFU/mL, a concentration that would theoretically allow the differences in mortality to be verified since it would not lead to 100% lethality in all groups. Furthermore, if any strains were to cause cardiotoxic effects, this inoculum would be enough to make them evident, as seen with strain ATCC 13883.

### 2.3. Zebrafish Virulence Model

**Figure 2:**
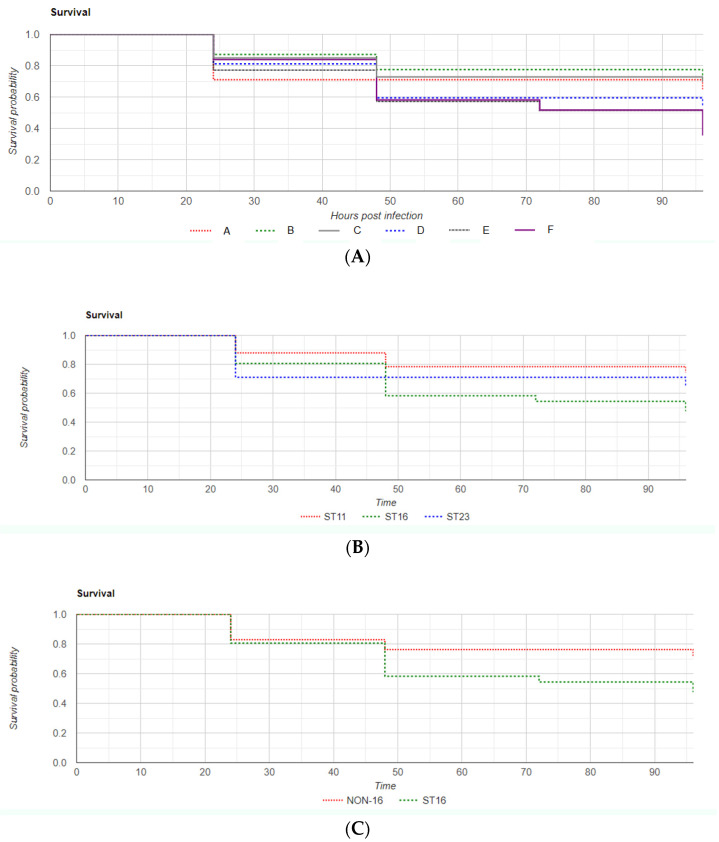
Overall zebrafish Kaplan–Meier survival curve (**A**) according *to K. pneumoniae* strain of infection; (**B**) Kaplan–Meier curves when grouping survival of embryos by ST of infection; (**C**) Kaplan–Meier survival curves comparing zebrafish infection by *K. pneumoniae* ST16 strains with non-ST16 strains. Time is expressed in hours post-infection (hpi).

In the virulence model, overall mortality rates at 96 h post infection were 42% (*n* = 84) for the bacterial strain injection groups and 4% (*n* = 2) for the control groups (χ^2^ = 28.9, *p* < 0.00001). Mortality rates at 24-, 48-, 72-, and 96-h post-infection for each strain are displayed in Table 2. Data are also presented as Kaplan–Meier survival rate curves, which can be found in Figure 2. A statistical analysis of data difference was performed by chi-squared log rank tests (χ^2^ = 8.17, *p* = 0.14).

When establishing sequence type as the variable of interest, there was statistical significance in mortality difference among zebrafish groups infected with ST16, ST11, and ST23 (52%, 26%, and 35%, respectively, log rank χ^2^ = 10.03, *p* = 0.007) (Figure 2B).

An analysis mortality data grouping ST16 vs. non-ST16 infected zebrafish revealed significantly higher mortality in the ST16 group (52% in ST16 vs. 29% in non-ST16, log rank χ^2^ = 9.19, *p* = 0.002) (Figure 2C).

Cardiotoxicity was observed at 96 hpi: 36% with strain “C”, 30% with “E”, 4% with “D”, and 3% with “A”. There was a significantly higher cardiotoxic effect with strains “C” and “E” (ST11 and ST16, respectively). Neither carry genes for virulence factors in common except for type 3 fimbriae, which is also expressed by other strains in this study.

The PCR for the *bla*_KPC-2_ gene was performed, with a positive result in all 6 bacteria-injected groups and a negative result in both control groups.

## 3. Discussion

We established the optimal inoculum concentration of *K. pneumoniae* harboring KPC-2 for zebrafish experiments as 10^6^ CFU/mL, a concentration that allowed the differences in mortality to be verified. We observed significantly higher mortality in the ST16-infected group compared with non-ST16 in the animal model.

Previous studies observed the association of ST16 and mortality. A study by Andrey et al. (2019) had previously reported a 95% mortality associated with ST16, which is a far higher mortality rate than what we report here, although both papers suffered from a lack of crucial clinical data. They also report findings from an infection model in *Galleria mellonella* showing a tendency for higher mortality in ST16-infected groups [11]. *Galleria mellonella* has been used as an infection model with *Enterobacteriaceae*, but there has been a recent criticism of the method [5]. zebrafish, on the other hand, present appealing qualities for infection models. *Danio rerio* is not able to express adaptive immunity components until it has reached its adult stage, which makes it an excellent model for assessing interactions between a pathogen and a host’s innate immunity in the early stages of the infectious process. In addition, its genome has already been completely annotated and is highly homologous to the human genome, a very attractive quality for animal models [12,13].

Our results found that ST16 strains have led to a greater number of deaths among zebrafish embryos than the other ST strains, with a statistically significant difference. Interestingly, the highest mortality in our experiment was due to strain “F”, an ST16 strain, even though it carries in its genome far fewer virulence-related genes than other strains in this study. *K. pneumoniae* “B”, for example, is an ST11 strain that possesses the genetic material to express several virulence factors that “F” cannot, such as stress adaptation, siderophores, and autotransporters. Strain “A”, on the other hand, belongs to ST23, a sequence type classically associated with a virulent phenotype, and the WGS found that it carried several virulence-related genes, such as the nutritional factor for allantoin metabolism, the *mnt*B stress-adaptation factor, and two types of iron-acquisition factors (Aerobactin, Yersiniabactin); in spite of that, it led to a very similar mortality rate in zebrafish embryos to the ones seen with ST11 strains that carry far fewer virulence factors. The ability to metabolize allantoin in *K. pneumoniae* has been linked to clinical presentation in the form of pyogenic liver abscesses; unlike *E. coli*, which is unable to rely on allantoin as a sole source of carbon, *K. pneumoniae* is able to use it as a sole source of both carbon and nitrogen, either in aerobic or anaerobic conditions, which may be related to higher virulence [14]. “A” was the only strain in our study theoretically able to metabolize allantoin.

However, other studies such as one conducted by Yu et al. (2007) in diabetes mellitus patients have demonstrated that serotype itself is not a predictor of hypervirulence (hvKp); while up to 70% of all hvKp present K1 or K2 capsule serotypes, those serotypes have also been widely observed in classic *K. pneumoniae* strains (cKp) and therefore cannot be accurately used for predicting virulence [15]. Some studies have demonstrated that other genetic markers are more sensitive in predicting the hvKp phenotype, such as the *iro* and *iuc* gene families (siderophores) and RmpA/RmpA2, regulatory genes related to the hyperexpression of capsule components [5,16]. In this study, such genetic components were scarcely found and were not predictors of virulence.

The limitation of our findings is that capsule serotype identification was not performed.

## 4. Materials and Methods

### 4.1. Bacterial Strains, Whole-Genome Sequencing, and Genetic Mapping

Forty-six strains of colistin-resistant *K. pneumoniae* were obtained from patients admitted to three tertiary teaching hospitals in Brazil (Hospital das Clínicas da Faculdade de Medicina de São Paulo-HCFMUSP/SP-2000 beds; Hospital Universitário de Londrina/PR-317 beds; Hospital Universitário de Santa Maria/RS-403 beds) between 2011 and 2019. These strains were also resistant to all beta-lactams (including carbapenems) and aminoglycosides or quinolones. Species identification was carried out by the Microbiology Division of the Central Laboratory of HCFMUSP using Matrix-Assisted Laser Desorption Ionization Time Of Flight Mass Spectrometry (MALDI-TOF, VITEK^®^ MS, bioMérieux-France), and preliminary susceptibility testing was performed using an automated method (VITEK-2 system, bioMérieux-France). Colistin resistance was confirmed through microdilution in cation-adjusted Müeller–Hinton II broth, according to Clinical and Laboratory Standards Institute (CLSI) M100 specifications [17]. In cases of multi-resistance, strains were also tested for susceptibility to Ceftazidime-Avibactam in Müeller–Hinton agar using Etest strips (bioMérieux-France) [18].

The isolates were stored in the strain collection located at a Medical Research Laboratory (LIM49) at the Institute of Tropical Medicine of the University of São Paulo. All were submitted to whole-genome sequencing (WGS) by a Illumina MiSeq (Illumina Inc., San Diego, CA, USA) next-generation sequencer.

*Klebsiella pneumoniae* strain MGH78578 (GenBank accession number CP000647.1) was used as a reference for molecular characterization. Multi-locus-sequence typing (MLST), virulome, and resistome analysis were performed using MLSTFinder 2.0 [19], VirulenceFinder 2.0 [20], VFDB [21], and ResFinder 4.0 [22], respectively.

### 4.2. Selection of Strains for the In Vivo Experimental Model

Isolates were selected for the animal study based on their sequence type and on possible genetic predictors of virulence found in WGS. Six strains were selected for the model, hereby identified by the letters A (strain 4223), B (strain 4290), C (strain 5921), D (strain 4449), E (strain 4553), and F (strain 5919). All of them harbor *bla*_KPC-2_; when considering MLST, strain A belongs to ST23, B and C belong to ST11, and strains D through F belong to ST16. The six patients from whom isolates were obtained were immunocompromised (five post hematopoietic stem cell transplantation and one acquired immunodeficiency syndrome), and strains were all obtained from bloodstream infection.

### 4.3. Zebrafish: Inoculum Determination and Virulence Model

Since data on experiments with bacterial inoculum in zebrafish are scarce and the size of the inoculum varied from 10^2^ to 10^8^ CFU/mL in the few studies that have been published [3,8,9,10], we opted for conducting a preliminary dose-response step in order to determine the best inoculum (lethal concentration 50; LC50) for comparing the six selected *K. pneumoniae* strains. The fish embryo acute toxicity test with zebrafish is designed to determine the acute toxicity of exogenous substances on embryonic stages of fish [23,24]. For this test, we used *K. pneumoniae* ATCC 13883, a common cKp strain used in microbiology, as a test control, at concentrations of 10^2^, 10^4^, 10^6^, 10^8^, and 10^9^ CFU/mL. ATCC 13883 carries only fimbriae as virulent factors in its genome, with no genes related to iron acquisition, allantoin metabolism, capsule, and regulation.

All procedures involving *Danio rerio* were approved by HCFMUSP’s Ethics Commission on Animal Use beforehand (CEUA submission number 1584/2020) on October, 2020. Viable zebrafish embryos were selected 48–72 h post-fertilization (hpf) and anesthetized in a culture medium with 0.1% tricaine. They were divided into seven groups, one for each inoculum concentration plus two control groups (one injected with saline and one with culture medium). Each group comprised approximately 30 embryos, distributed in seven plaques. All injections were performed in the embryos’ Cuvier’s ducts (caudal veins) and had a total volume of 4 nL of bacterial suspension. Embryos were evaluated every 24 h by a professional, with the final analysis at 96 h post infection (hpi). Two primary endpoints-viability and cardiac edema-plus other relevant findings upon microscope analysis were assessed.

As determined in the preliminary step and shown in the Section 2 of this article, the six previously selected strains (“A”, “B”, “C”, “D”, “E”, and “F”) were diluted in saline to a final concentration of 10^6^ CFU/mL and reserved for the experiment. Viable zebrafish embryos were selected 48–72 hpf and anesthetized in a culture medium with 0.1% tricaine. For our experimental virulence model, in total, 198 zebrafish embryos were injected with *K. pneumoniae* strains, and 55 embryos were control injected with either saline or culture medium. They were divided into eight groups, one for each strain to be tested plus two control groups (one injected with saline and one with culture medium). Each group comprised 30 embryos, distributed in eight plaques. All injections were performed in the embryos’ caudal veins with 4 nL of bacterial suspension (approximately four CFU per embryo), and, for a total of 96 h, embryos were evaluated as previously described [25].

After 96 h, the remaining viable fish embryos were euthanized with the addition of lethal doses of tricaine to culture medium, and DNA was extracted from all embryos. The DNA extraction of each pool of embryos (each corresponding to a different strain) was performed by reaction with proteinase K. To confirm the infection of embryos, all isolates were submitted to polymerase chain reaction (PCR) for resistance gene *bla*_KPC-2_ as previously described [26]. Since the complete sterility of fish cultures cannot be guaranteed, it was opted for *bla*_KPC-2_ as a marker gene instead of more generic ones, such as 16S rRNA. Data are shown as Kaplan–Meier survival curves with confidence intervals (CI) under Results; statistical analysis was conducted using the Statistics Kingdom Kaplan–Meier plotter, the log rank test calculator, and the chi-squared variance test calculator with a *p*-value significance of *p* < 0.05 [27].

## 5. Conclusions

In zebrafish embryos, *K. pneumoniae* strains belonging to ST16 are associated with significantly higher mortality when compared to other sequence types. The optimal inoculum concentration of *K. pneumoniae* harboring *bla*_KPC-2_ established for zebrafish experiments was 10^6^ CFU/mL. Further research is necessary to better understand the role of each specific virulence factor in the pathogenic process and the correlation between sequence type and virulence.

## Figures and Tables

**Table 1:** Virulence factors and ST of all six strains used for the zebrafish model, according to WGS.

Strain	MLST	Siderophore	Regulation	Allantoin Metabolism	Auto Transporter	Efflux Pump AcrAB	Stress Adaptation
“A”	23	Aerobactin, Yersiniabactin	*rcs*A, *rcs*B	Yes	No	Yes	Yes
“B”	11	Enterobactin, Yersiniabactin	*rcs*A, *rcs*B	No	*cah*	Yes	Yes
“C”	11	Enterobactin, Yersiniabactin	*rcs*A, *rcs*B	No	*cah*	Yes	Yes
“D”	16	None	*rcs*A, *rcs*B	No	No	Yes	No
“E”	16	None	*rcs*A, *rcs*B	No	No	Yes	No
“F”	16	Enterobactin, Yersiniabactin	*rcs*A, *rcs*B	No	No	Yes	No

**Table 2:** Survival (*n*) and partial mortality rates (PMR) of each group at 24-, 48-, 72-, and 96-h post-infection (hpi). Group IDs correspond to the strains used to infect embryos in each group.

Group ID	ST	Injected Embryos	Alive 24 hpi	PMR	Alive 48 hpi	PMR	Alive 72 hpi	PMR	Alive 96 hpi	Endpoint Mortality
“A”	23	31	22	29%	22	29%	22	29%	20	35%
“B”	11	31	27	13%	24	22%	24	22%	22	29%
“C”	11	33	28	15%	24	27%	24	27%	23	30%
“D”	16	37	30	19%	22	40%	22	40%	20	46%
“E”	16	35	27	23%	20	43%	18	48%	18	48%
“F”	16	31	26	16%	18	42%	16	48%	11	64%

## Data Availability

All sequenced strains were deposited on NCBI under the Bioproject accession number PRJNA377546.
